# 1-(5-Bromo-4-phenyl-1,3-thia­zol-2-yl)pyrrolidin-2-one

**DOI:** 10.1107/S160053681201954X

**Published:** 2012-05-16

**Authors:** Hazem A. Ghabbour, Adnan A. Kadi, Hussein I. El-Subbagh, Tze Shyang Chia, Hoong-Kun Fun

**Affiliations:** a Department of Pharmaceutical Chemistry, College of Pharmacy, King Saud University, PO Box 2457, Riyadh 11451, Saudi Arabia; b Department of Pharmaceutical Chemistry, Faculty of Pharmaceutical Sciences and Pharmaceutical Industries, Future University, Cairo 12311, Egypt; c X-ray Crystallography Unit, School of Physics, Universiti Sains Malaysia, 11800 USM, Penang, Malaysia

## Abstract

The asymmetric unit of the title compound, C_13_H_11_BrN_2_OS, consists of two crystallographically independent mol­ecules (*A* and *B*). In each mol­ecule, the pyrrolidine ring adopts an envelope conformation with a methyl­ene C atom as the flap atom. In mol­ecule *A*, the central thia­zole ring makes a dihedral angle of 36.69 (11)° with the adjacent phenyl ring, whereas the corresponding angle is 36.85 (12)° in mol­ecule *B*. The pyrrolidine ring is slightly twisted from the thia­zole ring, with C—N—C—N torsion angles of 4.8 (3) and 3.0 (4)° in mol­ecules *A* and *B*, respectively. In the crystal, C—H⋯π and π–π [centroid-to-centroid distance = 3.7539 (14) Å] inter­actions are observed. The crystal studied was a pseudo-merohedral twin with twin law (-100 0-10 101) and a refined component ratio of 0.7188 (5):0.2812 (5).

## Related literature

For background to thiazoles, see: Bishayee *et al.* (1997 ▶); Chitamber & Wereley (1997 ▶); Bhaskar *et al.* (2008 ▶); Sharma *et al.* (2009 ▶); Bhattacharya *et al.* (2005 ▶); Spector *et al.* (1998 ▶). For ring-puckering parameters, see: Cremer & Pople (1975 ▶). For the stability of the temperature controller used for data collection, see: Cosier & Glazer (1986 ▶).
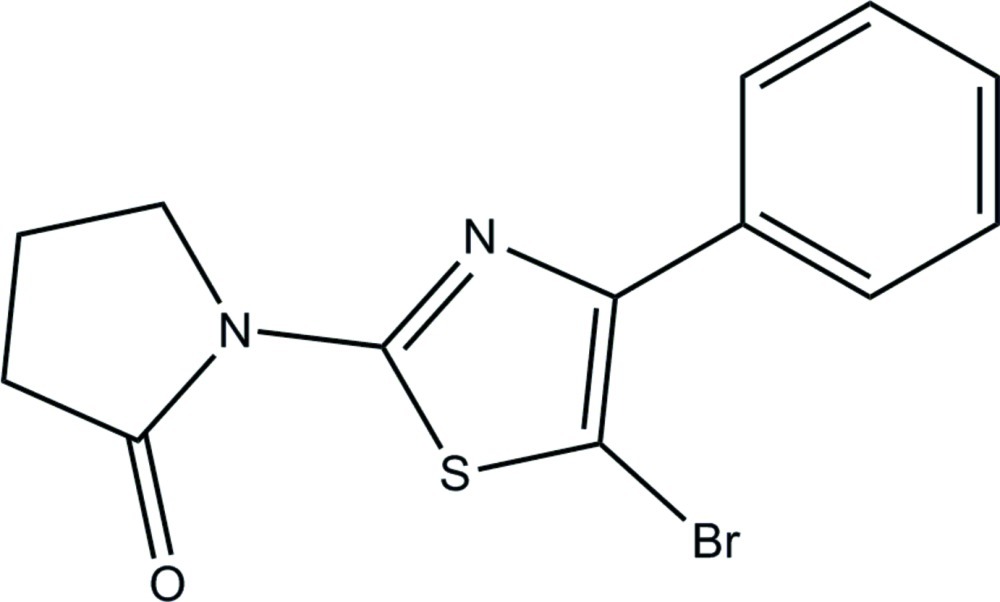


## Experimental

### 

#### Crystal data

C_13_H_11_BrN_2_OS
*M* *_r_* = 323.21
Monoclinic, 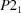

*a* = 7.5243 (3) Å
*b* = 14.1861 (6) Å
*c* = 12.4488 (6) Å
β = 107.508 (1)°
*V* = 1267.23 (10) Å^3^
*Z* = 4
Mo *K*α radiation
μ = 3.40 mm^−1^
*T* = 100 K
0.26 × 0.14 × 0.14 mm

#### Data collection

Bruker APEX DUO CCD area-detector diffractometer
Absorption correction: multi-scan (*SADABS*; Bruker, 2009 ▶) *T*_min_ = 0.466, *T*_max_ = 0.646
30838 measured reflections
9338 independent reflections
8701 reflections with *I* > 2σ(*I*)
*R*_int_ = 0.032

#### Refinement

*R*[*F* ^2^ > 2σ(*F* ^2^)] = 0.027
*wR*(*F* ^2^) = 0.051
*S* = 0.99
9338 reflections
326 parameters
1 restraint
H-atom parameters constrained
Δρ_max_ = 0.58 e Å^−3^
Δρ_min_ = −0.46 e Å^−3^
Absolute structure: Flack (1983 ▶), with 4219 Friedel pairs
Flack parameter: 0.017 (4)

### 

Data collection: *APEX2* (Bruker, 2009 ▶); cell refinement: *SAINT* (Bruker, 2009 ▶); data reduction: *SAINT*; program(s) used to solve structure: *SHELXTL* (Sheldrick, 2008 ▶); program(s) used to refine structure: *SHELXTL*; molecular graphics: *SHELXTL*; software used to prepare material for publication: *SHELXTL* and *PLATON* (Spek, 2009 ▶).

## Supplementary Material

Crystal structure: contains datablock(s) global, I. DOI: 10.1107/S160053681201954X/is5131sup1.cif

Structure factors: contains datablock(s) I. DOI: 10.1107/S160053681201954X/is5131Isup2.hkl

Supplementary material file. DOI: 10.1107/S160053681201954X/is5131Isup3.cml

Additional supplementary materials:  crystallographic information; 3D view; checkCIF report

## Figures and Tables

**Table 1:** Hydrogen-bond geometry (Å, °) *Cg*1 is the centroid of the C1*B*–C6*B* ring.

*D*—H⋯*A*	*D*—H	H⋯*A*	*D*⋯*A*	*D*—H⋯*A*
C12*A*—H12*B*⋯*Cg*1^i^	0.97	2.89	3.767 (3)	151

Symmetry code: (i) 
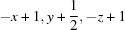
.

**Fig. 1.**
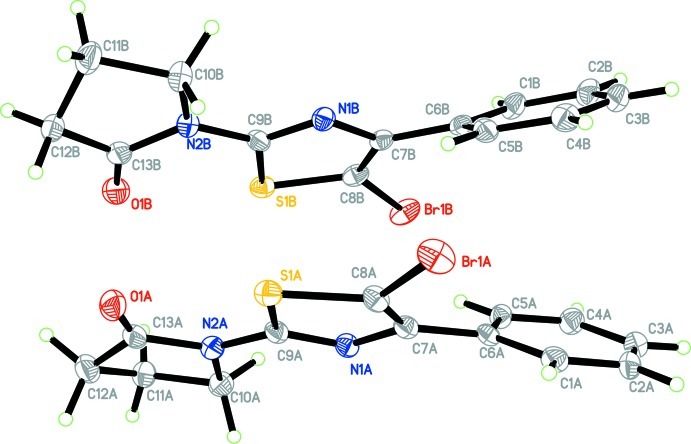
The asymmetric unit of the title compound with atom labels and 50% probability displacement ellipsoids.

## supplementary crystallographic information

### Comment

Thiazole is a five-membered ring system with two hetero atoms (S, N) placed at the 1 and 3 positions of the heterocycle. The nucleus is a building block in the structure of various natural products and biologically active compounds, like thiamine (vitamin-B), also in some antibiotics drugs like penicillin, micrococcin and many metabolic products of fungi and primitive marine animals (Bhaskar *et al.*, 2008). Thiazole-containing drugs have widespread use in a variety of medical conditions such as fungal and bacterial infections, gastric ulcers, cancer, *etc* (Bishayee *et al.*, 1997). Thiazole derivatives are involved frequently as the subject of drug design and synthesis efforts and they are reported to possess several activities like antibacterial, antifungal, anti-inflammatory (Sharma *et al.*, 2009), analgesic, antitubercular, central nervous system (CNS) stimmulant activity as well as anti-HIV activity (Bhattacharya *et al.*, 2005). Aminothiazole derivatives are well explored as agents of potential biological activities and some of the derivatives of thiazoles have shown inhibition towards herpes simplex virus (Spector *et al.*, 1998).

The asymmetric unit of the title compound (Fig. 1) consists of two crystallographically independent molecules (*A* and *B*). In both molecules, the pyrrolidine ring (N2/C10–C13) adopts an envelope conformation with atom C11 as the flap atom [puckering parameters (Cremer & Pople, 1975), *Q* = 0.272 (3) Å and φ = 254.4 (5)° in molecule *A*; *Q* = 0.282 (3) Å and φ = 74.7 (5)° in molecule *B*]. In molecule *A*, the central thiazole ring (S1/N1/C7–C9) makes a dihedral angle of 36.69 (11)° with the adjacent benzene ring (C1–C6), whereas the corresponding angle is 36.85 (12)° in molecule *B*. The pyrrolidine ring is slightly twisted from the thiazole ring with C10—N2—C9—N1 torsion angles of 4.8 (3) and 3.0 (4)° in molecules *A* and *B*, respectively.

In the crystal packing, no significant intermoelcular hydrogen bondings are observed. The crystal packing is stabilized by C—H···*Cg*1 and π—π [*Cg*2—*Cg*3 = 3.7539 (14) Å; symmetry code = 1-X,1/2+Y,1-Z] interactions, where *Cg*1, *Cg*2 and *Cg*3 are the centroids of C1B–C6B, S1A/N1A/C7A–C9A and S1B/N1B/C7B–C9B rings, respectively.

### Experimental

4-Chlorobutanoyl chloride (423 mg, 3 mmol) was added dropwise to a solution of 5-bromo-4-phenylthiazol-2-amine (255 mg, 1 mmol) and K_2_CO_3_ (414 mg, 3 mmol) in CHCl_3_. The mixture was stirred for 48 h at room temperature and then ammonia and water were added to remove excess 4-chlorobutanoyl chloride and K_2_CO_3_. The organic solvent was removed in vacuum. The residue was taken up in dry toluene and the solution was refluxed for 10 h after addition of excess amount of piperidine. The mixture was cooled and the solvent was removed in vacuum to give solid which was then purified by chromatotron and crystallized from ethanol to give the single crystals.

### Refinement

All H atoms were positioned geometrically (C—H = 0.93 and 0.97 Å) and refined using a riding model, with *U*_iso_(H) = 1.2*U*_eq_(C). Three outliers (-1 5 2), (0 2 0) and (-3 0 1) were omitted. The crystal was a twin with twin law (100 010 101) and BASF = 0.2812 (5).

### Crystal data

**Table:**

C_13_H_11_BrN_2_OS	*F*(000) = 648
*M**_r_* = 323.21	*D*_x_ = 1.694 Mg m^−^^3^
Monoclinic, *P*2_1_	Mo *K*α radiation, λ = 0.71073 Å
Hall symbol: P 2yb	Cell parameters from 9880 reflections
*a* = 7.5243 (3) Å	θ = 2.2–32.0°
*b* = 14.1861 (6) Å	µ = 3.40 mm^−^^1^
*c* = 12.4488 (6) Å	*T* = 100 K
β = 107.508 (1)°	Block, colourless
*V* = 1267.23 (10) Å^3^	0.26 × 0.14 × 0.14 mm
*Z* = 4	

### Data collection

**Table:**

Bruker APEX DUO CCD area-detector diffractometer	9338 independent reflections
Radiation source: fine-focus sealed tube	8701 reflections with *I* > 2σ(*I*)
Graphite monochromator	*R*_int_ = 0.032
φ and ω scans	θ_max_ = 33.6°, θ_min_ = 1.4°
Absorption correction: multi-scan (*SADABS*; Bruker, 2009)	*h* = −11→11
*T*_min_ = 0.466, *T*_max_ = 0.646	*k* = −21→21
30838 measured reflections	*l* = −19→19

### Refinement

**Table:**

Refinement on *F*^2^	Secondary atom site location: difference Fourier map
Least-squares matrix: full	Hydrogen site location: inferred from neighbouring sites
*R*[*F*^2^ > 2σ(*F*^2^)] = 0.027	H-atom parameters constrained
*wR*(*F*^2^) = 0.051	*w* = 1/[σ^2^(*F*_o_^2^) + (0.0136*P*)^2^] where *P* = (*F*_o_^2^ + 2*F*_c_^2^)/3
*S* = 0.99	(Δ/σ)_max_ = 0.001
9338 reflections	Δρ_max_ = 0.58 e Å^−^^3^
326 parameters	Δρ_min_ = −0.46 e Å^−^^3^
1 restraint	Absolute structure: Flack (1983), with 4219 Friedel pairs
Primary atom site location: structure-invariant direct methods	Flack parameter: 0.017 (4)

### Special details

**Table:**

Experimental. The crystal was placed in the cold stream of an Oxford Cryosystems Cobra open-flow nitrogen cryostat (Cosier & Glazer, 1986) operating at 100.0 (1) K.
Geometry. All e.s.d.'s (except the e.s.d. in the dihedral angle between two l.s. planes) are estimated using the full covariance matrix. The cell e.s.d.'s are taken into account individually in the estimation of e.s.d.'s in distances, angles and torsion angles; correlations between e.s.d.'s in cell parameters are only used when they are defined by crystal symmetry. An approximate (isotropic) treatment of cell e.s.d.'s is used for estimating e.s.d.'s involving l.s. planes.
Refinement. Refinement of *F*^2^ against ALL reflections. The weighted *R*-factor *wR* and goodness of fit *S* are based on *F*^2^, conventional *R*-factors *R* are based on *F*, with *F* set to zero for negative *F*^2^. The threshold expression of *F*^2^ > σ(*F*^2^) is used only for calculating *R*-factors(gt) *etc*. and is not relevant to the choice of reflections for refinement. *R*-factors based on *F*^2^ are statistically about twice as large as those based on *F*, and *R*-factors based on ALL data will be even larger.

### Fractional atomic coordinates and isotropic or equivalent isotropic displacement parameters (Å^2^)

**Table:**

	*x*	*y*	*z*	*U*_iso_*/*U*_eq_	
Br1A	1.01261 (3)	0.351681 (14)	0.70723 (2)	0.02637 (6)	
S1A	0.68748 (7)	0.33990 (4)	0.48703 (5)	0.01897 (10)	
O1A	0.4724 (3)	0.32508 (11)	0.26929 (13)	0.0249 (3)	
N1A	0.4385 (2)	0.34234 (16)	0.59269 (14)	0.0167 (3)	
N2A	0.3089 (3)	0.33380 (14)	0.39687 (16)	0.0189 (4)	
C1A	0.7366 (3)	0.39986 (15)	0.87748 (19)	0.0214 (4)	
H1AA	0.8276	0.4358	0.8601	0.026*	
C2A	0.7282 (3)	0.39718 (17)	0.98699 (19)	0.0243 (5)	
H2AA	0.8110	0.4331	1.0423	0.029*	
C3A	0.5979 (3)	0.34160 (19)	1.01478 (19)	0.0246 (5)	
H3AA	0.5954	0.3389	1.0890	0.030*	
C4A	0.4701 (3)	0.28953 (17)	0.93178 (19)	0.0225 (4)	
H4AA	0.3815	0.2524	0.9501	0.027*	
C5A	0.4761 (3)	0.29351 (16)	0.82224 (18)	0.0179 (4)	
H5AA	0.3905	0.2590	0.7667	0.022*	
C6A	0.6091 (3)	0.34881 (16)	0.79332 (17)	0.0162 (4)	
C7A	0.6099 (3)	0.34893 (16)	0.67534 (17)	0.0167 (4)	
C8A	0.7554 (3)	0.34945 (18)	0.63183 (17)	0.0176 (4)	
C9A	0.4600 (3)	0.33758 (15)	0.49273 (18)	0.0166 (4)	
C10A	0.1169 (3)	0.34222 (19)	0.40144 (19)	0.0225 (4)	
H10A	0.0897	0.4061	0.4195	0.027*	
H10B	0.0937	0.2992	0.4563	0.027*	
C11A	0.0021 (4)	0.31523 (16)	0.2810 (2)	0.0244 (5)	
H11A	−0.1123	0.3515	0.2568	0.029*	
H11B	−0.0288	0.2487	0.2762	0.029*	
C12A	0.1303 (3)	0.33887 (18)	0.20894 (19)	0.0267 (5)	
H12A	0.1118	0.2946	0.1471	0.032*	
H12B	0.1068	0.4022	0.1786	0.032*	
C13A	0.3242 (3)	0.33079 (14)	0.28908 (19)	0.0212 (4)	
Br1B	0.15444 (3)	0.079853 (14)	0.69491 (2)	0.02544 (5)	
S1B	0.24659 (7)	0.09024 (4)	0.46979 (4)	0.01913 (10)	
O1B	0.2343 (3)	0.11605 (13)	0.25040 (14)	0.0269 (4)	
N1B	0.6031 (2)	0.08191 (17)	0.56770 (14)	0.0179 (3)	
N2B	0.5265 (3)	0.09060 (15)	0.37119 (14)	0.0191 (3)	
C1B	0.5995 (3)	0.02734 (16)	0.85721 (18)	0.0220 (4)	
H1BA	0.4904	−0.0079	0.8416	0.026*	
C2B	0.7207 (4)	0.03050 (17)	0.96597 (19)	0.0253 (5)	
H2BA	0.6929	−0.0037	1.0226	0.030*	
C3B	0.8816 (3)	0.0835 (2)	0.99154 (18)	0.0260 (5)	
H3BA	0.9604	0.0860	1.0651	0.031*	
C4B	0.9255 (3)	0.13339 (18)	0.9064 (2)	0.0234 (5)	
H4BA	1.0339	0.1692	0.9229	0.028*	
C5B	0.8065 (3)	0.12933 (17)	0.79710 (18)	0.0188 (4)	
H5BA	0.8372	0.1619	0.7403	0.023*	
C6B	0.6420 (3)	0.07735 (17)	0.77091 (17)	0.0167 (4)	
C7B	0.5198 (3)	0.07758 (17)	0.65372 (15)	0.0160 (3)	
C8B	0.3295 (3)	0.0800 (2)	0.61500 (18)	0.0194 (4)	
C9B	0.4773 (3)	0.08761 (18)	0.47029 (16)	0.0170 (4)	
C10B	0.7195 (3)	0.0830 (2)	0.37033 (17)	0.0212 (4)	
H10C	0.7822	0.0302	0.4157	0.025*	
H10D	0.7884	0.1404	0.3975	0.025*	
C11B	0.6969 (4)	0.06701 (18)	0.2457 (2)	0.0272 (5)	
H11C	0.7981	0.0963	0.2246	0.033*	
H11D	0.6946	0.0002	0.2287	0.033*	
C12B	0.5114 (4)	0.11300 (18)	0.18401 (19)	0.0242 (5)	
H12C	0.5289	0.1781	0.1657	0.029*	
H12D	0.4496	0.0793	0.1151	0.029*	
C13B	0.4007 (3)	0.10679 (15)	0.2667 (2)	0.0219 (5)	

### Atomic displacement parameters (Å^2^)

**Table:**

	*U*^11^	*U*^22^	*U*^33^	*U*^12^	*U*^13^	*U*^23^
Br1A	0.01407 (9)	0.02869 (12)	0.03443 (13)	−0.00219 (9)	0.00438 (9)	0.00372 (11)
S1A	0.0193 (2)	0.0182 (2)	0.0212 (2)	0.0000 (2)	0.00885 (19)	0.0019 (2)
O1A	0.0317 (9)	0.0245 (8)	0.0207 (7)	−0.0017 (7)	0.0111 (7)	0.0014 (6)
N1A	0.0130 (8)	0.0190 (9)	0.0170 (8)	0.0014 (7)	0.0028 (6)	0.0004 (7)
N2A	0.0210 (9)	0.0174 (9)	0.0172 (8)	0.0011 (7)	0.0040 (7)	−0.0007 (7)
C1A	0.0223 (11)	0.0151 (9)	0.0232 (10)	−0.0031 (8)	0.0016 (9)	0.0011 (8)
C2A	0.0289 (12)	0.0215 (10)	0.0162 (10)	0.0000 (9)	−0.0029 (9)	−0.0020 (8)
C3A	0.0295 (11)	0.0239 (11)	0.0185 (10)	0.0055 (10)	0.0042 (9)	0.0003 (10)
C4A	0.0231 (11)	0.0249 (10)	0.0208 (11)	0.0014 (9)	0.0084 (9)	−0.0008 (9)
C5A	0.0158 (9)	0.0199 (10)	0.0170 (10)	−0.0001 (7)	0.0032 (8)	−0.0018 (7)
C6A	0.0134 (8)	0.0172 (9)	0.0162 (8)	0.0039 (8)	0.0015 (7)	0.0023 (8)
C7A	0.0154 (8)	0.0143 (8)	0.0186 (9)	−0.0006 (8)	0.0023 (7)	0.0007 (8)
C8A	0.0133 (8)	0.0187 (9)	0.0193 (9)	−0.0007 (9)	0.0024 (7)	0.0020 (9)
C9A	0.0151 (9)	0.0164 (8)	0.0171 (9)	0.0017 (8)	0.0031 (8)	0.0013 (7)
C10A	0.0180 (10)	0.0251 (11)	0.0212 (10)	0.0043 (9)	0.0013 (8)	−0.0024 (9)
C11A	0.0264 (12)	0.0250 (9)	0.0191 (10)	0.0000 (9)	0.0031 (9)	−0.0020 (9)
C12A	0.0322 (13)	0.0242 (11)	0.0193 (10)	−0.0004 (10)	0.0008 (9)	0.0020 (9)
C13A	0.0292 (11)	0.0148 (9)	0.0180 (9)	0.0006 (8)	0.0047 (10)	0.0016 (7)
Br1B	0.02277 (10)	0.02499 (10)	0.03492 (12)	−0.00498 (9)	0.01830 (9)	−0.00685 (11)
S1B	0.0166 (2)	0.0177 (2)	0.0224 (2)	0.0005 (2)	0.00493 (19)	−0.0016 (2)
O1B	0.0266 (9)	0.0261 (8)	0.0232 (8)	0.0029 (7)	0.0002 (7)	−0.0029 (7)
N1B	0.0194 (8)	0.0191 (7)	0.0175 (7)	0.0016 (9)	0.0092 (6)	0.0007 (8)
N2B	0.0216 (9)	0.0206 (8)	0.0159 (7)	0.0007 (8)	0.0068 (7)	0.0003 (7)
C1B	0.0279 (11)	0.0214 (10)	0.0198 (10)	−0.0028 (9)	0.0117 (9)	−0.0028 (8)
C2B	0.0368 (13)	0.0242 (11)	0.0183 (11)	0.0021 (9)	0.0134 (10)	−0.0002 (8)
C3B	0.0294 (12)	0.0310 (11)	0.0174 (9)	0.0074 (12)	0.0067 (8)	−0.0033 (11)
C4B	0.0180 (10)	0.0266 (11)	0.0262 (12)	0.0014 (9)	0.0075 (9)	−0.0016 (9)
C5B	0.0176 (10)	0.0220 (11)	0.0191 (10)	0.0051 (8)	0.0089 (9)	0.0024 (8)
C6B	0.0214 (9)	0.0134 (8)	0.0182 (9)	0.0021 (9)	0.0104 (7)	−0.0008 (9)
C7B	0.0190 (8)	0.0132 (7)	0.0189 (8)	0.0000 (8)	0.0105 (7)	0.0000 (8)
C8B	0.0200 (9)	0.0165 (8)	0.0246 (10)	−0.0014 (9)	0.0113 (8)	−0.0022 (10)
C9B	0.0171 (9)	0.0168 (8)	0.0178 (9)	0.0027 (8)	0.0061 (7)	0.0037 (9)
C10B	0.0200 (10)	0.0269 (10)	0.0168 (9)	−0.0025 (11)	0.0058 (7)	−0.0016 (11)
C11B	0.0349 (13)	0.0291 (13)	0.0215 (10)	−0.0019 (10)	0.0146 (10)	−0.0015 (9)
C12B	0.0358 (13)	0.0206 (10)	0.0168 (10)	0.0016 (10)	0.0086 (9)	−0.0012 (8)
C13B	0.0285 (12)	0.0156 (10)	0.0195 (10)	0.0018 (8)	0.0042 (9)	−0.0019 (7)

### Geometric parameters (Å, º)

**Table:**

Br1A—C8A	1.880 (2)	Br1B—C8B	1.874 (2)
S1A—C8A	1.725 (2)	S1B—C8B	1.732 (2)
S1A—C9A	1.7349 (19)	S1B—C9B	1.734 (2)
O1A—C13A	1.214 (3)	O1B—C13B	1.214 (3)
N1A—C9A	1.304 (3)	N1B—C9B	1.297 (3)
N1A—C7A	1.390 (3)	N1B—C7B	1.395 (2)
N2A—C9A	1.379 (3)	N2B—C13B	1.379 (3)
N2A—C13A	1.381 (3)	N2B—C9B	1.391 (3)
N2A—C10A	1.468 (3)	N2B—C10B	1.460 (3)
C1A—C2A	1.384 (3)	C1B—C2B	1.387 (3)
C1A—C6A	1.392 (3)	C1B—C6B	1.402 (3)
C1A—H1AA	0.9300	C1B—H1BA	0.9300
C2A—C3A	1.381 (4)	C2B—C3B	1.379 (4)
C2A—H2AA	0.9300	C2B—H2BA	0.9300
C3A—C4A	1.393 (3)	C3B—C4B	1.393 (4)
C3A—H3AA	0.9300	C3B—H3BA	0.9300
C4A—C5A	1.379 (3)	C4B—C5B	1.387 (3)
C4A—H4AA	0.9300	C4B—H4BA	0.9300
C5A—C6A	1.401 (3)	C5B—C6B	1.393 (3)
C5A—H5AA	0.9300	C5B—H5BA	0.9300
C6A—C7A	1.471 (3)	C6B—C7B	1.472 (3)
C7A—C8A	1.360 (3)	C7B—C8B	1.367 (3)
C10A—C11A	1.536 (3)	C10B—C11B	1.526 (3)
C10A—H10A	0.9700	C10B—H10C	0.9700
C10A—H10B	0.9700	C10B—H10D	0.9700
C11A—C12A	1.539 (4)	C11B—C12B	1.522 (4)
C11A—H11A	0.9700	C11B—H11C	0.9700
C11A—H11B	0.9700	C11B—H11D	0.9700
C12A—C13A	1.504 (3)	C12B—C13B	1.509 (4)
C12A—H12A	0.9700	C12B—H12C	0.9700
C12A—H12B	0.9700	C12B—H12D	0.9700
			
C8A—S1A—C9A	86.83 (10)	C8B—S1B—C9B	87.07 (10)
C9A—N1A—C7A	110.89 (17)	C9B—N1B—C7B	110.50 (17)
C9A—N2A—C13A	123.6 (2)	C13B—N2B—C9B	123.5 (2)
C9A—N2A—C10A	121.85 (18)	C13B—N2B—C10B	114.04 (18)
C13A—N2A—C10A	114.25 (19)	C9B—N2B—C10B	122.35 (17)
C2A—C1A—C6A	120.1 (2)	C2B—C1B—C6B	119.8 (2)
C2A—C1A—H1AA	119.9	C2B—C1B—H1BA	120.1
C6A—C1A—H1AA	119.9	C6B—C1B—H1BA	120.1
C3A—C2A—C1A	120.6 (2)	C3B—C2B—C1B	121.1 (2)
C3A—C2A—H2AA	119.7	C3B—C2B—H2BA	119.4
C1A—C2A—H2AA	119.7	C1B—C2B—H2BA	119.4
C2A—C3A—C4A	120.1 (2)	C2B—C3B—C4B	119.6 (2)
C2A—C3A—H3AA	120.0	C2B—C3B—H3BA	120.2
C4A—C3A—H3AA	120.0	C4B—C3B—H3BA	120.2
C5A—C4A—C3A	119.4 (2)	C5B—C4B—C3B	119.6 (2)
C5A—C4A—H4AA	120.3	C5B—C4B—H4BA	120.2
C3A—C4A—H4AA	120.3	C3B—C4B—H4BA	120.2
C4A—C5A—C6A	121.0 (2)	C4B—C5B—C6B	121.2 (2)
C4A—C5A—H5AA	119.5	C4B—C5B—H5BA	119.4
C6A—C5A—H5AA	119.5	C6B—C5B—H5BA	119.4
C1A—C6A—C5A	118.8 (2)	C5B—C6B—C1B	118.7 (2)
C1A—C6A—C7A	122.8 (2)	C5B—C6B—C7B	118.49 (19)
C5A—C6A—C7A	118.37 (19)	C1B—C6B—C7B	122.8 (2)
C8A—C7A—N1A	112.61 (18)	C8B—C7B—N1B	113.14 (17)
C8A—C7A—C6A	130.06 (18)	C8B—C7B—C6B	128.69 (17)
N1A—C7A—C6A	117.22 (18)	N1B—C7B—C6B	118.04 (17)
C7A—C8A—S1A	113.25 (15)	C7B—C8B—S1B	112.33 (15)
C7A—C8A—Br1A	129.24 (16)	C7B—C8B—Br1B	129.92 (16)
S1A—C8A—Br1A	117.40 (11)	S1B—C8B—Br1B	117.67 (11)
N1A—C9A—N2A	121.45 (18)	N1B—C9B—N2B	121.12 (18)
N1A—C9A—S1A	116.41 (15)	N1B—C9B—S1B	116.93 (15)
N2A—C9A—S1A	122.11 (16)	N2B—C9B—S1B	121.95 (15)
N2A—C10A—C11A	102.29 (19)	N2B—C10B—C11B	102.22 (17)
N2A—C10A—H10A	111.3	N2B—C10B—H10C	111.3
C11A—C10A—H10A	111.3	C11B—C10B—H10C	111.3
N2A—C10A—H10B	111.3	N2B—C10B—H10D	111.3
C11A—C10A—H10B	111.3	C11B—C10B—H10D	111.3
H10A—C10A—H10B	109.2	H10C—C10B—H10D	109.2
C10A—C11A—C12A	104.3 (2)	C12B—C11B—C10B	104.71 (19)
C10A—C11A—H11A	110.9	C12B—C11B—H11C	110.8
C12A—C11A—H11A	110.9	C10B—C11B—H11C	110.8
C10A—C11A—H11B	110.9	C12B—C11B—H11D	110.8
C12A—C11A—H11B	110.9	C10B—C11B—H11D	110.8
H11A—C11A—H11B	108.9	H11C—C11B—H11D	108.9
C13A—C12A—C11A	104.47 (19)	C13B—C12B—C11B	103.98 (18)
C13A—C12A—H12A	110.9	C13B—C12B—H12C	111.0
C11A—C12A—H12A	110.9	C11B—C12B—H12C	111.0
C13A—C12A—H12B	110.9	C13B—C12B—H12D	111.0
C11A—C12A—H12B	110.9	C11B—C12B—H12D	111.0
H12A—C12A—H12B	108.9	H12C—C12B—H12D	109.0
O1A—C13A—N2A	123.3 (2)	O1B—C13B—N2B	123.8 (2)
O1A—C13A—C12A	129.6 (2)	O1B—C13B—C12B	129.3 (2)
N2A—C13A—C12A	107.1 (2)	N2B—C13B—C12B	106.9 (2)
			
C6A—C1A—C2A—C3A	2.2 (3)	C6B—C1B—C2B—C3B	1.2 (4)
C1A—C2A—C3A—C4A	−1.7 (4)	C1B—C2B—C3B—C4B	−1.3 (4)
C2A—C3A—C4A—C5A	0.5 (4)	C2B—C3B—C4B—C5B	0.2 (4)
C3A—C4A—C5A—C6A	0.2 (3)	C3B—C4B—C5B—C6B	1.0 (3)
C2A—C1A—C6A—C5A	−1.4 (3)	C4B—C5B—C6B—C1B	−1.1 (3)
C2A—C1A—C6A—C7A	179.7 (2)	C4B—C5B—C6B—C7B	178.7 (2)
C4A—C5A—C6A—C1A	0.2 (3)	C2B—C1B—C6B—C5B	0.0 (3)
C4A—C5A—C6A—C7A	179.1 (2)	C2B—C1B—C6B—C7B	−179.8 (2)
C9A—N1A—C7A—C8A	0.4 (3)	C9B—N1B—C7B—C8B	0.3 (3)
C9A—N1A—C7A—C6A	−176.2 (2)	C9B—N1B—C7B—C6B	−175.9 (2)
C1A—C6A—C7A—C8A	38.0 (4)	C5B—C6B—C7B—C8B	−141.2 (3)
C5A—C6A—C7A—C8A	−140.9 (3)	C1B—C6B—C7B—C8B	38.6 (4)
C1A—C6A—C7A—N1A	−146.1 (2)	C5B—C6B—C7B—N1B	34.3 (3)
C5A—C6A—C7A—N1A	35.1 (3)	C1B—C6B—C7B—N1B	−145.9 (2)
N1A—C7A—C8A—S1A	−1.0 (3)	N1B—C7B—C8B—S1B	−1.3 (3)
C6A—C7A—C8A—S1A	175.1 (2)	C6B—C7B—C8B—S1B	174.4 (2)
N1A—C7A—C8A—Br1A	−176.95 (19)	N1B—C7B—C8B—Br1B	−177.9 (2)
C6A—C7A—C8A—Br1A	−0.8 (4)	C6B—C7B—C8B—Br1B	−2.2 (4)
C9A—S1A—C8A—C7A	0.9 (2)	C9B—S1B—C8B—C7B	1.4 (2)
C9A—S1A—C8A—Br1A	177.41 (16)	C9B—S1B—C8B—Br1B	178.51 (17)
C7A—N1A—C9A—N2A	−177.8 (2)	C7B—N1B—C9B—N2B	−178.5 (2)
C7A—N1A—C9A—S1A	0.3 (3)	C7B—N1B—C9B—S1B	0.9 (3)
C13A—N2A—C9A—N1A	178.5 (2)	C13B—N2B—C9B—N1B	−172.8 (2)
C10A—N2A—C9A—N1A	4.8 (3)	C10B—N2B—C9B—N1B	3.0 (4)
C13A—N2A—C9A—S1A	0.5 (3)	C13B—N2B—C9B—S1B	7.8 (3)
C10A—N2A—C9A—S1A	−173.23 (18)	C10B—N2B—C9B—S1B	−176.4 (2)
C8A—S1A—C9A—N1A	−0.7 (2)	C8B—S1B—C9B—N1B	−1.4 (2)
C8A—S1A—C9A—N2A	177.4 (2)	C8B—S1B—C9B—N2B	178.1 (2)
C9A—N2A—C10A—C11A	−169.7 (2)	C13B—N2B—C10B—C11B	−16.4 (3)
C13A—N2A—C10A—C11A	16.0 (3)	C9B—N2B—C10B—C11B	167.4 (2)
N2A—C10A—C11A—C12A	−25.5 (2)	N2B—C10B—C11B—C12B	26.6 (3)
C10A—C11A—C12A—C13A	26.6 (2)	C10B—C11B—C12B—C13B	−27.8 (3)
C9A—N2A—C13A—O1A	4.9 (3)	C9B—N2B—C13B—O1B	−3.2 (4)
C10A—N2A—C13A—O1A	179.1 (2)	C10B—N2B—C13B—O1B	−179.3 (2)
C9A—N2A—C13A—C12A	−173.4 (2)	C9B—N2B—C13B—C12B	175.1 (2)
C10A—N2A—C13A—C12A	0.8 (3)	C10B—N2B—C13B—C12B	−1.0 (3)
C11A—C12A—C13A—O1A	164.4 (2)	C11B—C12B—C13B—O1B	−163.6 (2)
C11A—C12A—C13A—N2A	−17.4 (2)	C11B—C12B—C13B—N2B	18.2 (3)

### Hydrogen-bond geometry (Å, º)

*Cg*1 is the centroid of the C1B–C6B ring.

**Table:**

*D*—H···*A*	*D*—H	H···*A*	*D*···*A*	*D*—H···*A*
C12*A*—H12*B*···*Cg*1^i^	0.97	2.89	3.767 (3)	151

Symmetry code: (i) −*x*+1, *y*+1/2, −*z*+1.
